# X‐ray Excited Optical Fluorescence and Diffraction Imaging of Reactivity and Crystallinity in a Zeolite Crystal: Crystallography and Molecular Spectroscopy in One

**DOI:** 10.1002/ange.201601796

**Published:** 2016-05-04

**Authors:** Zoran Ristanović, Jan P. Hofmann, Marie‐Ingrid Richard, Tao Jiang, Gilbert A. Chahine, Tobias U. Schülli, Florian Meirer, Bert M. Weckhuysen

**Affiliations:** ^1^ Debye Institute for Nanomaterials ScienceUtrecht UniversityUniversiteitsweg 993584 CGUtrechtThe Netherlands; ^2^ Department of Chemical Engineering and ChemistryEindhoven University of Technology, P.O. Box 5135600 MBEindhovenThe Netherlands; ^3^ European Synchrotron Radiation Facility6 rue Jules Horowitz38043Grenoble CedexFrance; ^4^ Aix Marseille Université, CNRS, Université de Toulon, IM2NP UMR 7334, 13397MarseilleFrance

**Keywords:** Chemische Bildgebung, Heterogene Katalyse, Röntgenbeugung, Zeolithe, ZSM-5

## Abstract

Structure–activity relationships in heterogeneous catalysis are challenging to be measured on a single‐particle level. For the first time, one X‐ray beam is used to determine the crystallographic structure and reactivity of a single zeolite crystal. The method generates μm‐resolved X‐ray diffraction (μ‐XRD) and X‐ray excited optical fluorescence (μ‐XEOF) maps of the crystallinity and Brønsted reactivity of a zeolite crystal previously reacted with a styrene probe molecule. The local gradients in chemical reactivity (derived from μ‐XEOF) were correlated with local crystallinity and framework Al content, determined by μ‐XRD. Two distinctly different types of fluorescent species formed selectively, depending on the local zeolite crystallinity. The results illustrate the potential of this approach to resolve the crystallographic structure of a porous material and its reactivity in one experiment via X‐ray induced fluorescence of organic molecules formed at the reactive centers.

## Graphical abstract

**Lokale Kristallinität** und säurekatalysierte Reaktionen in einem Zeolith‐Einkristall wurden mit Mikroröntgenbeugung (μ‐XRD) und optischer Fluoreszenz nach Anregung fluoreszierender Reaktionsprodukte (μ‐XEOF) mit einem Röntgen‐Strahl ermittelt. Verwachsungen und die Al‐Verteilung sind entscheidend für das Maß an Entaluminierung und die formselektive Produktbildung.

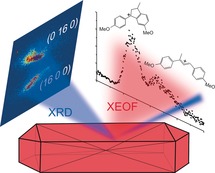

Zeolites are microporous aluminosilicates that play a major role as solid acid catalysts in industries.^1^, ^2^, ^3^ Zeolite framework aluminum is commonly related to the catalytically active Brønsted acid sites.^4^, ^5^ The single crystal architecture and distribution of Al sites over short‐ and long‐range distances^6^ influence the overall catalytic activity and success of various post‐treatment methods aiming to improve mass transport by controlled dealumination and desilication.^7^, ^8^, ^9^

A remarkable example of the compositional and structural complexity of zeolites is ZSM‐5 with the MFI topology, often found with pronounced Al zoning^10^, ^11^, ^12^, ^13^ and complex internal intergrowth structures.^14^, ^15^ Both Al zoning and architecture of the crystals may strongly affect the outcome of post‐synthesis modifications and lead to remarkable differences in mesoporosity^8^, ^16^ and reactivity.^17^, ^18^ Whereas various micro‐spectroscopy methods previously introduced provided a wealth of information about inter‐ and intra‐particle heterogeneities in structure and reactivity,^15^, ^19^, ^20^, ^21^ direct structure–reactivity relationships remain difficult to establish.

Herein, we present a novel characterization approach based on synchrotron micro‐X‐ray diffraction (μ‐XRD) imaging combined with μ‐X‐ray excited optical fluorescence (μ‐XEOF) imaging used to obtain a spatially resolved, structure–performance relationship of a single zeolite ZSM‐5 crystal. We exploit the full potential of X‐rays^22^, ^23^, ^24^, ^25^, ^26^, ^27^, ^28^, ^29^ by using one X‐ray beam to acquire both diffraction and spectral information. The local zeolite crystallinity, as measured by μ‐XRD, is correlated with the local presence of Brønsted acidity, as measured by μ‐XEOF.

For this study we used large zeolite ZSM‐5 crystals^18^, ^30^, ^31^, ^32^ and a Brønsted acid‐catalyzed probe reaction based on the oligomerization of 4‐methoxystyrene. Upon the protonation of 4‐methoxystyrene on zeolite ZSM‐5, oligomeric carbocations are formed, revealing the location of accessible Brønsted acid sites.^20^, ^32^ If excited by X‐rays, these molecules undergo photoemission in the optical region (UV/Vis), a phenomenon that is generally known as X‐ray excited optical luminescence,^33^, ^34^, ^35^ here referred to as XEOF. Recently, several strategies were developed at synchrotrons to utilize the XEOF emission of visible light for studies of functional materials.^36^, ^37^, ^38^, ^39^, ^40^, ^41^, ^42^, ^43^, ^44^ Our method makes use of a less common method to simultaneously excite electronic transitions in organic molecules and resolve the crystallographic structure of a single crystal.

Figure 1 illustrates the approach for measuring μ‐XRD/μ‐XEOF maps of a single steamed ZSM‐5 crystal stained with 4‐methoxystyrene in one experiment. Details of the setup can be found in the Supporting Information. Hard X‐rays (8.5 keV) focused to a spot size of 500 nm were used for the successive μ‐XRD and μ‐XEOF imaging of a single ZSM‐5 crystal. Figure 1 a shows the response of an X‐ray detector for specific (16 0 0) and (0 16 0) Bragg reflections, which were previously used to study the intergrowth structure of zeolite ZSM‐5.^13^ An optical fiber for the collection of the XEOF signal was placed in the close proximity to the sample stage at ca. 200–300 μm distance (Supporting Information, Figure S1). The X‐ray excitation of the formed cyclic and linear dimeric styrene species takes place along the beam trajectory, which results in a XEOF spectrum (Figure 1 b). The resulting fluorescence is related to the accessible and reactive Brønsted acid sites, where the formation of the fluorescent species takes place.

**Figure 1:**
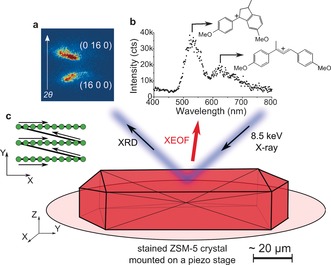
The μ‐XRD/μ‐XEOF experiment for measuring a steamed ZSM‐5 crystal stained with 4‐methoxystyrene. a) Response of the 2D X‐ray detector upon detection of the characteristic (16 0 0) and (0 16 0) Bragg reflections, where the arrow indicates the direction of the scattering angle, 2*θ*. b) XEOF spectrum detected with the UV/Vis spectrograph with the indicated emission bands of cyclic and linear dimeric species. c) X‐Y scanning pattern used to acquire spatially resolved μ‐XRD/μ‐XEOF intensity maps.

The evaluation of the XEOF signal with fluorescence microscopy and the inherent photobleaching processes of the fluorescent carbocations in the presence of X‐rays are described in the Supporting Information. The recorded fluorescence intensity decayed with the time constant of 4.5±0.3 s (Supporting Information, Figure S2). The photobleaching showed clear dose‐dependent behavior but did not cause the formation of new fluorescent bands. Prior to the 2D XEOF scans, the beam damage to organic molecules was minimized by using neutral density filters. Acquisition time of 1.95 s per point was chosen to collect good quality XEOF spectra and avoid potential artefacts that are due to photobleaching. As a compromise between the sampling frequency and scanning time, the spatially correlated maps were acquired in steps of 4 μm for μ‐XEOF (1.95 s exposure time, ET) and 2 μm for diffraction (20–50 ms ET) with the typical X‐Y scanning pattern presented in Figure 1 c. The diffraction rocking maps were collected after the μ‐XEOF intensity maps (a single X‐Y scan), by changing the incident angle of the beam and repeating X‐Y scans for 13 rocking angles, in steps of Δ*θ*=0.1°. The X‐Y scans were carried out in a fast scanning mode (K‐map), as described by Chahine et al.^45^

To evaluate the impact of steaming on reactivity, we investigated a steamed ZSM‐5 crystal with a more complex intergrowth structure (Figure 2 a). The 90° intergrowth of the selected ZSM‐5 crystal seems to be interconnected in an anomalous manner, when compared to previous reports.^14^, ^46^ The spatial distribution of the crystallographic phases was resolved by integrating the contributions of the higher‐order (16 0 0) and (0 16 0) Bragg reflections for a given range of X,Y positions (Figure 2 b). The resulting spatially resolved diffraction maps, obtained in X‐ray strain orientation calculation software (XSOCS),^45^ reveal the anomalous and asymmetrical crystal growth (Figure 2 c). The contribution of each phase in the diffraction signal will depend on the orientation of the phase with respect to the optical path of the X‐ray beam (Figure 2 d).

**Figure 2:**
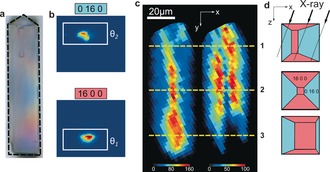
μ‐XRD imaging of the ZSM‐5 crystal with a complex intergrowth structure. a) Optical micrograph of the crystal; the dashed black line indicates an irregular shape of the crystal. b) Typical X‐ray detector responses for the studied (16 0 0) and (0 16 0) reflections, positioned at Bragg angles of *θ*_1_ and *θ*_2_. c) Spatial distribution of the diffraction signals for (16 0 0) reflection (left) and (0 16 0) reflection (right) as calculated by XSOCS software.^45^ The diffraction intensities were summed over all 13 rocking angles for the regions of interest defined in (b). The yellow lines denote the vertical (X‐Z) cross‐sections shown in (d). d) Exposure of the different crystallographic subunits along the optical path of an X‐ray beam; the dotted lines illustrate the propagation of the X‐ray beam throughout the crystal resulting in the diffraction information from different crystalline domains.

The ZSM‐5 crystal was tested for XEOF response in the visible region by collecting X‐ray excited fluorescence light during a raster scan of the crystal. An averaged XEOF spectrum summed over all collected data points is shown in Figure 3 a. An intense emission band with the highest intensity in all recorded XEOF spectra appeared at about 530 nm, followed by two less intense emission bands at circa 615 nm and circa 670 nm. The latter two bands appeared to be red‐shifted (up to 20 nm) as compared to the fluorescence microscopy spectra (600 and 650 nm) of the same species (Supporting Information, Figure S3). These two emission bands have been previously attributed to linear dimeric and trimeric species that are confined along the straight pores of ZSM‐5.^20^, ^32^, ^47^ The higher‐energy XEOF band at 530 nm is assigned to cyclic dimeric species. Unlike the lower‐energy bands, the band at 530 nm was not detected in the μ‐XEOF experiments with parent zeolite crystals (Supporting Information, Figure S3). Furthermore, Fornes et al.^48^ and Stavitski et al.^47^ have reported the UV/Vis absorption band at 490 nm originating from cyclic dimeric carbocations. The same species are found to be formed at the near‐surface acid sites and crystalline defects induced by steaming.^49^, ^50^

**Figure 3:**
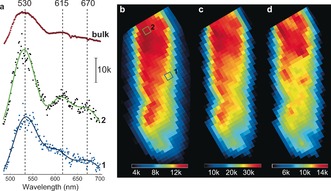
μ‐XEOF imaging of the steamed ZSM‐5 crystal. a) Examples of XEOF spectra: spectra 1 and 2 are taken from the regions indicated in (b); top: total XEOF spectrum averaged over all measured spectra. b) Averaged μ‐XEOF total intensity map; color bar: average number of counts per pixel. c) μ‐XEOF intensity map at 530±5 nm. d) μ‐XEOF intensity map at 610±10 nm; c) and d) are plotted based on the amplitudes of the fitted Gaussians.

Figure 3 a presents two XEOF spectra taken from different positions along the crystal (1 and 2 in Figure 3 b). We note that the position and intensity of the emission maximum between 610–615 nm change depending on the extent of reactivity. The emission maximum was about 615 nm for the highly reactive domains (spectrum 2, Figure 3 a), and shifted towards higher energies (600 nm) for the domains with lower XEOF intensity (spectrum 1, Figure 3 a). We attribute the observed shift to intermolecular interactions of the closely packed oligomeric carbocations.^32^, ^33^

The 2D μ‐XEOF map in Figure 3 b shows a notable gradient in XEOF intensity towards the bottom side of the crystal. Clearly, steaming has unevenly affected different parts of the crystal. To resolve the differences in the positions and amplitudes of the emission bands we have applied a Gaussian deconvolution of the XEOF spectra, by fitting the XEOF spectra with three Gaussians centered at the emission maxima at 530, 610, and 670 nm. The spatially resolved maps of the XEOF intensities are shown in Figure 3 c for the cyclic species and Figure 3 d for the linear dimeric species.

The vertical positions of the studied reflections on the X‐ray detector can be translated into the corresponding 2*θ* values (Figure 1 a). In this way, X‐ray diffractograms were constructed as 1D representations of the 2D detector response. To illustrate the complexity of the μ‐XRD/μ‐XEOF data set, seven different points were chosen along the crystal (Figure 4 a) to show both the recorded X‐ray diffractograms (Figure 4 b) and corresponding XEOF spectra (Figure 4 c). Principal component analysis (PCA) and subsequent clustering turned out to be very powerful to classify the recorded data sets according to their spectral feature. Analysis of the μ‐XRD data set divided the 2D diffraction intensity map into five clusters that have distinct diffraction features, which are represented by different colors in the PCA‐XRD map (Figure 4 d). Similar classification was made with the μ‐XEOF data set (Figure 4 e). The color‐coded diffractograms and XEOF spectra in Figure 4 b,c highlight spectral differences between the individual clusters in Figure 4 d,e, respectively. Averaged cluster spectra of both PCA‐XRD and PCA‐XEOF clusters are shown in the Supporting Information, Figure S4.

**Figure 4:**
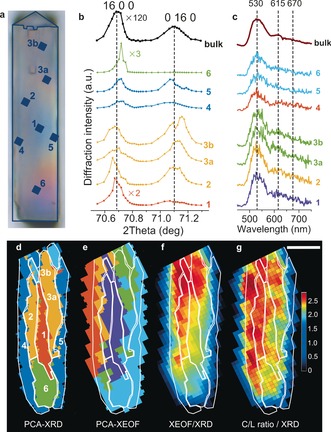
a) Optical micrograph of the steamed ZSM‐5 crystal with the positions of the sampling points. b) Diffractograms of the regions of interest labeled in (a). The color‐coding corresponds to the PCA‐XRD clusters presented in (d). c) XEOF spectra of the regions of interest labeled in (a). The color‐coding corresponds to the PCA‐XEOF clusters presented in (e). d) PCA cluster map of the μ‐XRD data set. The numbers denote the points used in (b) and (c). e) PCA cluster map of the μ‐XEOF data set overlaid with the contours of the XRD clusters. f,g) Overlay of the PCA‐XRD clustered regions from (d) and the μ‐XEOF intensity maps for the cyclic species (f) and a cyclic‐to‐linear intensity ratio (g). The color bar indicates the XEOF intensity ratio; the scale bar is 20 μm.

PCA divided the 2D map of crystallinity based on the intensities and positions of the (16 0 0) and (0 16 0) reflections, which translate directly into the strain in the crystal lattice that is imposed by Al enrichment/depletion. The outer regions of the crystal (blue clusters, points 4 and 5 in Figure 4 b) show low XRD peak intensities; the (0 16 0) peak is notably shifted towards higher d‐spacings (lower 2*θ* values) as observed previously for parent zeolite crystals,^13^ whereas a maximum of the (16 0 0) peak seems to be shifted towards lower d‐spacings. In a parent ZSM‐5 crystal, the outer region is Al‐rich^13^ and subsequent steaming leads to dealumination and contraction of the unit cell along the *a* lattice vector. The overlay of the PCA‐XRD clustered regions and XEOF intensity map in Figure 4 f indicates the lowest XEOF intensity, meaning the lowest reactivity, in these clusters. μ‐XRD intensity maps in Figure 2 c suggest the highest diffraction intensity originating from the middle of the crystal. The diffractograms of the inner clusters, depicted in orange (points 2, 3a,b) and red (point 1) in Figure 4 b,d, confirm the higher content of framework Al and a lower degree of dealumination. Consequently, their reactivity is higher, as visible from the most intense XEOF emission from the inner regions in Figure 4 f. A very distinct feature is the green cluster (point 6 in Figure 4 b,d) that represents a highly crystalline domain with the lowest d‐spacing for the (16 0 0) reflection, which is an indication of the Al‐poor phase that is more resistant to steaming and less reactive due to lower accessibility of the microcrystalline domains.

The XEOF intensity ratio of the cyclic and linear dimeric species can be used as an indication of the extent of reactivity that is determined by crystallinity and accessibility of the zeolite domains, as shown in the intensity map of this ratio (Figure 4 g). This map resembles the PCA‐μ‐XEOF map from Figure 4 e, highlighting the differences in the XEOF spectra compared in Figure 3 a. The higher amount of cyclic dimeric species with respect to linear dimeric species correlates well with the total XEOF intensity and the loss of crystallinity in the ZSM‐5 crystal.

Our experimental approach in combination with PCA shows that a 2D μ‐XRD mapping can provide useful crystallographic information about the 3D structure of a single zeolite crystal. This is possible owing to the presence of 90° intergrowths and pronounced Al zoning that divide the analyzed volume into distinct crystallographic phases, visible also in PCA cluster maps (Figure 4 d). Although precise 3D information cannot be extracted from our measurements, the positions and orientations of different crystallographic phases can still be identified based on the previous knowledge of the crystallographic and compositional anisotropy within the parent crystals, as illustrated with the intergrowth model in Figure 2 d.^13^ The observed 2D zones of different crystallinity would not be present if the crystals would consist of a single homogeneous phase.

As a result of the described crystal anisotropy, crystallographic phases within one zeolite crystal may be unevenly affected by steaming and result in distinctly different reactivity. During steaming, the outer Al‐rich phase is more prone to dealumination than the inner Al‐poor crystalline domains, which is the direct consequence of the local Al concentration that affects the dealumination rate.^51^, ^52^, ^53^ In recent work, we have measured higher catalytic turnover rates in the inner regions of steamed ZSM‐5 crystals and detected severe dealumination and clustering of Al atoms at the surface of the crystals.^54^ It is important to note that the crystal lattice of a parent ZSM‐5 expands at the outer rim in both a and b directions due to Al zoning,^13^ with the lattice parameters of *a=*20.10±0.02 Å and *b=*19.92±0.02 Å. Upon steaming these parameters change to *a=*20.03±0.02 Å and *b=*19.93±0.02 Å. A 0.07 Å contraction in the *a* lattice parameter implies a crystallographic change and dealumination along the sinusoidal pores. HR‐SEM and FIB‐SEM studies by Karwacki, Aramburo et al. noticed higher susceptibility of sinusoidal pores towards steaming and the unidirectional nature of mesopores along the sinusoidal channels.^30^, ^31^

In summary, we have demonstrated that hard X‐rays can be used to acquire information from both X‐ray and visible spectral regions when studying the impact of the crystalline structure and mesoporous defects on Brønsted reactivity of a zeolite crystal in a single X‐ray shot. The study demonstrates that the intra‐particle differences in zeolite reactivity are determined by the underlying local crystalline structure and composition. Such important structure–reactivity relationships are difficult to derive from other characterization approaches; hence the developed method has the potential to substantiate, synchronously, in space and time, the structural and reactivity properties of many other important functional materials.

## Supporting information

As a service to our authors and readers, this journal provides supporting information supplied by the authors. Such materials are peer reviewed and may be re‐organized for online delivery, but are not copy‐edited or typeset. Technical support issues arising from supporting information (other than missing files) should be addressed to the authors.

Supplementary
