# Supplementary material for: X‐ray Excited Optical Fluorescence and Diffraction Imaging of Reactivity and Crystallinity in a Zeolite Crystal: Crystallography and Molecular Spectroscopy in One
Source: Angew Chem Weinheim Bergstr Ger. 2016 May 4;128(26):7622–6. doi: 10.1002/ange.201601796 (PMC4950131; doi:10.1002/ange.201601796)
Supplement: Supplementary file 1 — Supplementary [file ANGE-128-7622-s001.pdf]

Angewandte Chemie
Eine Zeitschrift der Gesellschaft Deutscher Chemiker

Supporting Information

X-ray Excited Optical Fluorescence and Diffraction Imaging of Reactivity and Crystallinity in a Zeolite Crystal: Crystallography and Molecular Spectroscopy in One

*Zoran Ristanović, Jan P. Hofmann, Marie-Ingrid Richard, Tao Jiang, Gilbert A. Chahine, Tobias U. Schülli, Florian Meirer, and Bert M. Weckhuysen**

ange_201601796_sm_miscellaneous_information.pdf

S1. Preparation of the steamed zeolite ZSM-5 crystals

The preparation procedure followed the work of Aramburo *et al*.[1] Templated zeolite ZSM-5 crystals were provided by ExxonMobil. The organic TPA template molecules were removed by a careful calcination (1 K/min) at 823 K for 8 h. After the template removal, the zeolite crystals were converted into their acidic form by a triple ion-exchange with 10 wt% ammonium nitrate (99+ %, Acros Organics) at 353 K, followed by 6 h calcination (2 K/min) at 773 K to release ammonia attached to the acid sites. The sample after this treatment is denoted as parent zeolite ZSM-5 and this material is further used for the preparation of steamed zeolite ZSM-5 crystals. Prior to steaming, parent zeolite ZSM-5 crystals were preheated to 393 K (5 K/min) for 60 min in a quartz tubular oven (Thermoline 79300) and heated further to 973 K at a heating rate of 5 K/min. Further steaming treatment was performed using water saturated (373 K) N_2 flow (150 ml/min) for 5 h.

S2. Staining of zeolite ZSM-5 crystals

4-methoxystyrene (97%, Sigma-Aldrich) was used as a probe molecule for performing the oligomerization reaction over Brønsted acid sites in the zeolite ZSM-5 crystals. The zeolite crystals were exposed to 4-methoxystyrene vapor for 4 h in a closed glass container at room temperature.

S3. Experimental details

The experiments were performed at beamline ID01 of the European Synchrotron Radiation Facility (ESRF) in Grenoble, France. A scheme of the experimental set-up as well as photographs of the diffractometer and sample stage are shown in Figure S1. Hard X-rays (8.5 keV) focused to a spot size of 500 nm were used for the simultaneous X-ray diffraction (XRD) and excited optical fluorescence (XEOF) imaging of a labeled zeolite ZSM-5 single crystal. The footprint of the beam was projected throughout the whole crystalline volume, with approximately 20 μm optical path in the axial direction. The emitted fluorescence light was collected via a 0.2 mm optical fiber and directed to a 300 mm focal length Czerny-Turner spectrograph (Andor Shamrock 300i) equipped with 150 lines/mm grating and a Peltier cooled highly sensitive EM-CCD camera (Andor Newton 970). The optical fiber was attached

to the sample stage of the goniometer via a separate piezo X,Y,Z positioning stage with a travel range of 100 μm in each direction. Once the collection of light was optimized, the fiber was kept in fixed geometry with respect to a selected individual zeolite crystal. An additional CCD camera, connected to a long-distance objective, was used to detect the weak fluorescence light and to align the X-ray beam with respect to the selected zeolite crystal and the optical fiber for XEOF collection. The diffracted X-rays were collected under Bragg conditions using a 2-D Maxipix X-ray detector in Bragg-Brentano diffraction geometry. The characteristic (16 0 0) and (0 16 0) reflections of single zeolite crystals were detected prior to a 2-D scan. Neutral density filters were used to minimize X-ray beam damage before XEOF mapping.

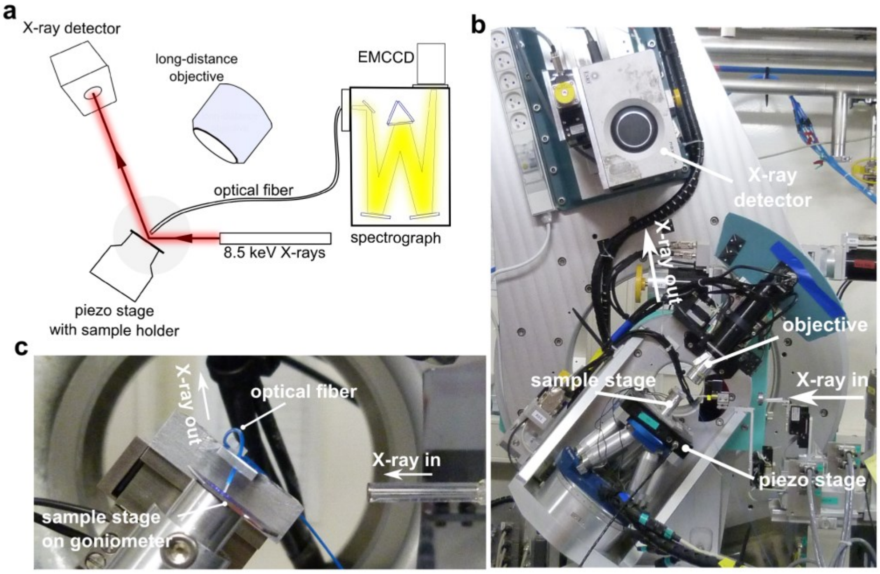

Figure S1. a) Schematic of the experimental setup used for the combined μ-XRD/μ-XEOF study. X-rays were focused down to a 500 nm spot to excite styrene oligomers and generate visible fluorescence collected with the optical fiber; diffracted X-rays were detected by the X-ray detector. b) Photograph of the setup showing the real geometry of the diffraction experiment. c) Zoom-in into the sample stage with the optical fiber attached.

Combined 2-D μ-XEOF and μ-XRD maps were generated using a piezo driven scanning stage in x-y direction. The X-ray beam and the exposure time of the detector were synchronized with an external trigger, resulting in 2 s of excitation with X-ray beam and 1.95 s of acquisition time of XEOF signal per collection point. The collected XEOF signal was further enhanced by full vertical binning and additional horizontal binning of 4 adjacent pixels at high electron-multiplier gain of the EMCCD camera. During 2-D XEOF scans both the crystal and optical fiber were kept in the fixed relative

position on the sample stage, thus the collection efficiency of the fiber was constant during experiments. A raster scan with piezo steps of typically 4 μm in X and Y was performed for the collection of XEOF intensity maps. The μ-XRD intensity maps were collected by using fast diffraction scanning (K-map) developed at ID01, as described by Chahine et al.[2] 2-D XRD mapping was typically performed in steps of 2 μm and 20-50 ms per collection point. The diffraction rocking maps were further collected by changing the incident angle of the beam and repeating X–Y scans for 10-15 rocking angles in fast scanning mode. The μ-XRD/μ-XEOF maps have been taken during separate scans, but directly after each other and without any sample or setup modifications. The main reason for the described scanning sequence is necessity of rocking scans for diffraction analysis. We have also performed a proof-of-principle experiment by synchronously collecting both X-ray diffractogram and XEOF emission using one X-ray beam. This approach was, however, avoided due to the limited information from X-ray diffraction at one angle.

S4. Properties of the XEOF signal

During inelastic scattering of X-rays, a process that is dominant for the case of interaction between hard X-rays and organic molecules, a large number of photo- and Auger electrons thermalize in the solid via cascade energy loss. These processes eventually lead to a radiative recombination of electrons (in LUMO orbitals) and holes (in HOMO orbitals) of organic molecules.

The origin and photo-properties of the XEOF signal were determined in a series of control experiments. 4-methoxystyrene readily colors zeolite ZSM-5 crystals deep purple at room temperature and the reaction appeared to be a convenient staining method for Brønsted acid sites. Unlabeled zeolite crystals, both in the templated and acidic form, did not show any emission of the XEOF signal in the absence of probe molecules.

Figure S2a presents a spatially resolved XEOF intensity map for a stained parent zeolite ZSM-5 crystal. The intensity map reflects a large heterogeneity in reactivity that is a consequence of structural defects visible in the optical image of the crystal (Figure S2b). The XEOF response matches well with the recorded wide-field fluorescence and laser induced confocal fluorescence microscopy

map of the same zeolite ZSM-5 crystal (Figures S2c). We note a small spatial broadening of the fluorescence intensities from the XEOF intensity map, which originates from the geometry of the μ-XEOF experiment and relatively large scanning steps (4 μm per acquisition point). Based on the remarkable spatial and spectral similarities between the μ-XEOF and confocal fluorescence microscopy results, we conclude that the oligomeric carbocationic species, formed upon reaction at Brønsted acid sites, are responsible for the observed XEOF signal.

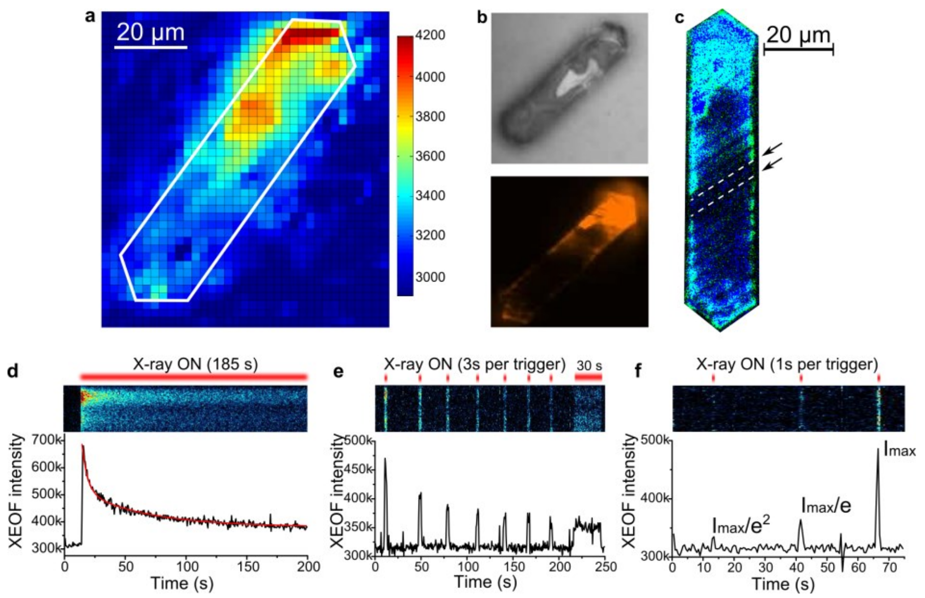

Figure S2. Properties of the XEOF emission and related photobleaching. a) μ-XEOF image of a parent ZSM-5 crystal stained with 4-methoxystyrene in the emission range of 600-750 nm. Color bar: averaged counts per wavelength channel. b) Wide-field optical (top) and fluorescence image (bottom) of the zeolite crystal. c) Confocal fluorescence microscopy image of the crystal acquired by using 561 nm excitation laser light, recorded 7 days after the XEOF measurement. The parallel dark stripes indicate irreversible beam damage; the arrows indicate the direction of an X-ray beam. d) Integrated intensity decay of the XEOF signal with constant exposure to X-rays, as measured on an agglomerate of steamed zeolite ZSM-5 crystals. The exponential fit (red line) has a time constant of t = 4.5 ± 0.3 s. e) Integrated intensity decay of the XEOF signal with the 3 s long pulsed exposure to the X-ray beam. f) XEOF signal response to X-rays of different intensity, with 1 s of the beam exposure. Spectral maps at the top of (d-f) represent the response of the detector for denoted exposure times. The red lines indicate that the beam was switched on.

High-energy X-rays turned out to cause irreversible photobleaching to the organic probe molecules, as it was confirmed later by additional confocal fluorescence microscopy measurements. The parallel stripes in Figure S2c indicate the direction and damage caused by the X-ray beam. The

photobleaching may be considered localized as the mean-free path of photo-electrons defined by their thermalization path (estimated to be in orders of 10 nm) is too small as compared to the size of the X-ray beam. Under the studied experimental conditions, the XEOF emission was characterized by the exponential decay of the fluorescence signal, with the decay time constant of 4.5 ± 0.3 s (Figure S2d). Although the decay of the XEOF signal was irreversible, the photo-bleaching of the XEOF emission did not affect our results, as the beam was sampling a new point in space every 2 s. The decay of the XEOF signal exhibited a clear dose-dependent behavior, as it was affected by the exposure time (Figure S2e) and the intensity of the X-ray beam (Figure S2f). The measured XEOF yield turned out to be intrinsically low, as only the highest achievable intensity of X-rays (order of 10^9 photons per s) was sufficient to generate a reasonable quality of XEOF spectra.

Finally, we compared the conventional confocal fluorescence microscopy spectra of the oligomeric species with the recorded XEOF spectra. This is illustrated in Figure S3 for the parent zeolite ZSM-5 crystal stained with 4-methoxystyrene. The absence of the cyclic dimeric species (~ 530 nm) was noted in both spectra and has been confirmed by scanning several parent crystals. Trimeric species are often found to easily form on parent crystals and their concentration is lower in steamed zeolite crystals, most probably due to lower probability for the successive oligomerization.[3] XEOF emission bands typically show a bathochromic shift of approximately 20 nm.

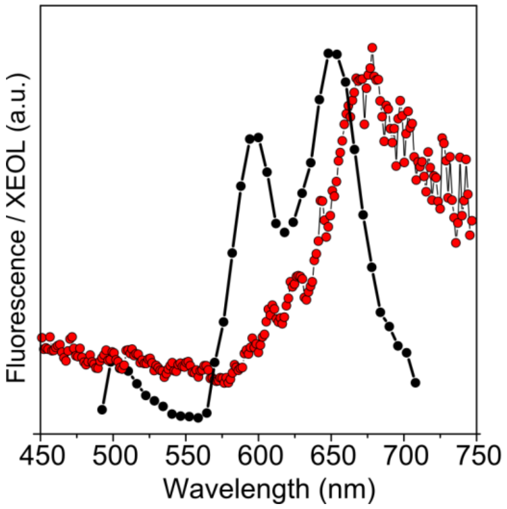

Figure S3. Comparison of the XEOF (red dots) and conventional confocal fluorescence microscopy spectra (black dots) of the linear dimeric (~ 600-625 nm) and trimeric carbocationic species (~ 650-675 nm) formed in purely microporous, parent zeolite ZSM-5 crystals; the XEOF spectrum was measured after 45 min of the gas-

phase staining with 4-methoxystyrene. The spectra are recorded from different crystals – the intensities of the emission bands may differ from crystal to crystal.

S5 Data treatment

The information from both μ-XRD and μ-XEOF data sets originates from identical crystalline regions along the optical path of the X-ray beam; however, the interpretation of the recorded data is additionally complicated due to: i) probing only one of the two 90° crystallographic orientations in μ-XRD and both orientation in μ-XEOF, ii) Al zoning along the crystal, and iii) uneven accessibility of different crystalline domains.

The 2-D μ-XEOF intensity maps were created by using home-build routines in *Matlab 2013a (MathWorks)*. For each X,Y collection point, a full XEOF spectrum was acquired and its integration (or deconvolution) within defined wavelengths, resulted in μ-XEOF intensity maps. Diffraction intensity maps were reconstructed by using XSOCS software (http://sourceforge.net/projects/xsocs/) written in the Python script language and developed at ESRF for the analysis of fast diffraction scanning data. A graphical interface for XSOCS has been developed using the PyGTK library.[2] The diffraction response within a defined 2θ region of interest was integrated over all incident angles as a function of X,Y coordinates of the piezo stage, yielding μ-XRD intensity maps.

Single-pixel XEOF spectra were deconvoluted by using simple Gaussian functions, fitted to the XEOF spectra in curve fitting toolbox (*Matlab R2013a*). Three Gaussian functions were centered at (530 ± 10) nm, (600 ± 20) nm, and (675 ± 5) nm with corresponding full widths at half maximum of (60 ± 8) nm, (50 ± 8) nm, and (67 ± 16) nm, respectively. An optimization procedure was applied to the data set by varying the positions and widths of Gaussian functions within the defined boundaries until the best fits were reached.

Principal component analysis (PCA) was performed on both the XEOF and the XRD data sets. PCA reduces the dimensionality of the data space by describing the data set in a way which best explains the total data's variance. This was done whilst preserving the relevant information and without using any *a priori* knowledge about the characteristics of the variations.[4,5] By choosing the amount of variance covered by the first N principal components (cumulative variance explained, CVE)

it was possible to reduce the dimensionality of the data set to fewer dimensions without losing significant information, but effectively reducing noise. The reduced data set was then represented in a N-dimensional PC space where k-means clustering[5] was performed to pool pixels according to their (Euclidean) distances from cluster centers (centroid linkage method), effectively grouping pixels with similar spectra. This resulted in an effective image segmentation based on the spectral similarity of the measurement points. Many of the original dimensions – that are typically not orthonormal - contain redundant information in terms of spectral similarity between pixels (i.e. similarity in spectral features such as peak positions, peak widths, peak heights etc.). By transforming the data to PC space we can therefore significantly reduce the number of PC's (*n*) necessary to describe the data without loosing significant information (i.e. variance in spectral features). Naturally, pixels with similar spectra will be located in close proximity in this new coordinate system. This is also true for the original coordinate system (*I* spectrum intensities) but the orthogonality of the PC space enhances spectral differences and therefore pattern in the scatter plot of the data (where every pixel is a data point in *I* dimensional space). A second advantage is the reduction of data dimensionality from *I* to *n*, because clustering is significantly faster in lower dimensional space.

The XEOF data set was constructed based on separate 2-D images each recorded at a specific wavelength channel. The 1-D diffractograms at each X,Y point were reconstructed by integration of the X-ray detector response with steps of 0.015° – 0.05° in 2θ. Therefore both PCA data sets consisted of a XEOF spectrum or XRD diffractogram in each point of the raster scan. Each data set formed a matrix ($I \times p$) where *I* denotes the intensity in the spectrum and *p* the pixel in the image. With each of those data matrices PCA and subsequent k-means clustering was performed separately to identify common spectral features and their distribution within the sample. The PCA-XRD and PCA-XEOF cluster images and average spectra of individual clusters are shown in Figure S4. Our analysis showed that even domains belonging to the same clusters can have different diffractograms and XEOF spectra because the principal component is dominated by the overall intensity of the spectra, which represents the strongest feature in the data (such as the regions #3a and #3b in Figure 4). Note, that the average spectra of clusters depend on the resolution of the clustering (i.e. the number of clusters used) and will

not necessarily provide pure chemical/crystallographic phases, but characteristic similarities in the spectra, more specifically, intensity ratios of the peaks and peak shifts.

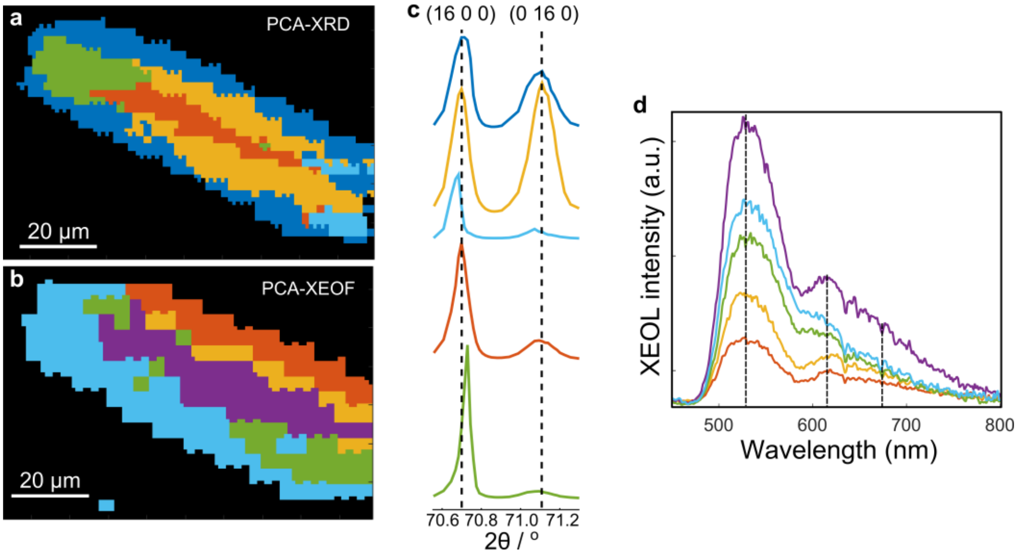

Figure S4. a) PCA-XRD cluster map of the distinct crystalline regions of steamed ZSM-5 crystal presented in Figure 4d of the main manuscript. b) PCA cluster map of the XEOF data set, presented in Figure 4e of the main manuscript. c) Averaged diffractograms of the individual color-coded clusters from (a). d) Averaged XEOF emission spectra of the individual color-coded clusters from (b)

Supporting references

[1] L. R. Aramburo, L. Karwacki, P. Cubillas, S. Asahina, D. A. M. de Winter, M. R. Drury, I. L. C. Buurmans, E. Stavitski, D. Mores, M. Daturi, P. Bazin, P. Dumas, F. Thibault-Starzyk, J. A. Post, M. W. Anderson, O. Terasaki, B. M. Weckhuysen, *Chem. – Eur. J.* **2011**, *17*, 13773–13781.

[2] G. A. Chahine, M.-I. Richard, R. A. Homs-Regojo, T. N. Tran-Caliste, D. Carbone, V. L. R. Jacques, R. Grifone, P. Boesecke, J. Katzer, I. Costina, H. Djazouli, T. Schroeder, T. U. Schülli, *J. Appl. Crystallogr.* **2014**, *47*, 762–769.

[3] M. H. F. Kox, E. Stavitski, J. C. Groen, J. Pérez-Ramírez, F. Kapteijn, B. M. Weckhuysen, *Chem. – Eur. J.* **2008**, *14*, 1718–1725.

[4] I. T. Jolliffe, *Principal Component Analysis*, Springer, New York, **2004**.

[5] J. E. Jackson, *A User's Guide to Principal Components*, Wiley-Interscience, Hoboken, **2003**.

[6] J. MacQueen, in *Proceedings of 5-Th Berkeley Symposium on Mathematical Statistics and Probability*, University Of California, **1967**, pp. 281–297.
